# Kissing Bond Damage Identification and Evaluation in CFRP-Reinforced Steel Plates Using Mixed-Frequency Ultrasonic Guided Waves

**DOI:** 10.3390/s26051531

**Published:** 2026-02-28

**Authors:** Ruiqi Guan, Haifeng Li, Weilong Ni, Tansheng Huang, Kai Wang, Xue Han

**Affiliations:** 1 College of Civil Engineering, Huaqiao University, Xiamen 361021, China; ruiqi.guan@hqu.edu.cn; 2 Xiamen Holsin Engineering Testing Co., Ltd., Xiamen 361027, China; 3 School of Aerospace Engineering, Xiamen University, Xiamen 361005, China; 4 School of Architecture and Civil Engineering, Xiamen Institute of Technology, Xiamen 361021, China

**Keywords:** mixed frequency response, nonlinear ultrasonic guided waves, CFRP-reinforced steel structures, kissing bond damage

## Abstract

CFRP laminates are widely adopted for the strengthening of steel structures and the debonding damage poses a severe threat to the integrity of CFRP-reinforced structures. However, as the early stage of debonding damage, kissing bond detection in these structures using the conventional ultrasonic guided waves method is a significant challenge due to the imperceptibility of microscale damage and the complexity of the wave properties at the interface. To address this problem, mixed-frequency ultrasonic guided waves with nonlinear characteristics are proposed to identify and evaluate kissing bond damage with different damage sizes in CFRP-reinforced steel structures. A finite element model is developed to simulate a kissing bond in a CFRP-reinforced steel plate and is utilized to investigate the interaction between mixed-frequency guided waves and the interface. Experimental tests are also carried out to verify the kissing bond detection method. Nonlinear parameters calculated based on the damage-induced sum and difference frequency components are employed to quantitatively evaluate the kissing bond damage. In addition, excitations with different wave modes are used in damage detection to compare their sensitivities to kissing bond damage. Both the simulation and experimental results reveal that the nonlinear parameter rises as the length of the kissing bond increases, reflecting the effectiveness of mixed-frequency ultrasonic guided wave for the identification and evaluation of kissing bond damage in CFRP-bonded structures.

## 1. Introduction

Steel structures are extensively utilized in civil infrastructure, such as in bridges, high-rise buildings and railway tracks. However, in-service steel structures may suffer from different types of damage, including corrosion pits and fatigue cracks, under hazardous environments and over a prolonged service time. In these cases, taking appropriate repair and retrofitting measures is essential to ensure the reliability and safety of the steel structure. With the advantages of its light weight and high strength, CFRP has been widely used to repair and strengthen damaged steel structures. Attaching to the steel components with an adhesive agent such as epoxy, CFRP increases the stiffness and bearing capacity of the whole structure, which in turn improves the service life of the aging steel structure [1]. The bonding condition between the CFRP and steel plate is a dominant factor in the performance of CFRP-reinforced steel structures. Thus, among all the failure modes of CFRP-reinforced structures, the major problem that threatens their performance is debonding between the CFRP and the steel components [2]. This sort of damage is generally caused by (a) overloading or fatigue load during the service period [3], (b) harsh environmental influence [4], and (c) improper installation [5].

To efficiently detect debonding in CFRP-reinforced structures, many non-destructive testing methods have been employed, including optical fiber technology [6,7,8], eddy current thermography [9,10], and ultrasonic guided waves [11,12,13]. The ultrasonic guided wave-based method, showing high sensitivity and high stability, has attracted many researchers’ interest. Ultrasonic guided waves with linear characteristics have been widely studied for the detection of interfacial debonding between composite materials and engineering structures. Zima et al. [11] investigated the propagation characteristics of ultrasonic guided waves in composite-bonded steel structures with different debonding configurations. Li et al. [14] used guided wave technology to monitor the growth of debonding damage in CFRP-reinforced steel plates based on correlation coefficient analysis. Chen et al. [15] carried out further studies on the influence of the position and degree of the debonding damage on the propagating waves. The imaging of debonding damage in CFRP-reinforced steel structures employing ultrasonic guided waves has also been proposed by many researchers based on different methods, including the weighted root mean square method [16] and the scanning probabilistic imaging method [5]. In addition, machine learning (e.g., the improved Elman neural network) has been combined with ultrasonic guided wave detection to predict debonding in CFRP-reinforced steel plates [17].

On many occasions, it is necessary to detect the debonding at the early stage efficiently before it deviates and causes structural failure, resulting in catastrophic accidents. As the early stage of debonding in CFRP reinforced structures, kissing bond is defined as intimate mechanical contact between the adhesive and adherend counterparts [18]. The sensitivity of the conventional ultrasonic testing method based on linear characteristics of guided waves, for example, the change of amplitudes and arrival time of received signals, is limited by the wavelength of the propagating wave. Kissing bond damage is a kind of micro-scale damage and cannot induce any changes in the linear characteristics of guided waves. Therefore, it is difficult to identify kissing bond damage using the conventional ultrasonic guided wave detection method. When a wave passes through the kissing bond interface, the contact interface would open and close under tensile and compressional phases of the wave, which is called breathing behavior, and would induce contact acoustic nonlinearity (CAN) [19] in the propagating waves. Based on this phenomenon, guided waves with nonlinear characteristics can be used to detect kissing bonds in structures. Different approaches based on nonlinear ultrasonic guided waves have been employed to detect the CAN caused by kissing bond damage in composite structures, including higher harmonic generation, subharmonic generation, mixed frequency response, etc. Li et al. [20] provided the image of debonding damage in CFRP-reinforced steel structures using higher harmonic generation approaches. Similar methods have been used on evaluation of bonding strength in more complicated multilayer composite structures, like pipeline coating [21]. Studies have proved that the mixed frequency response has shown less disturbance by the system nonlinearities, which can otherwise obscure the damage-induced CAN in the higher harmonic generation method [22]. Therefore, the mixed frequency method has also been employed to identify and image the debonding in FRP-strengthened concrete beam [23,24]. These studies have experimentally proved the detection capability of nonlinear guided waves on kissing bond damage in composite structures.

However, due to the anisotropic properties of CFRP and the apparent different material properties of the multilayer system, the wave propagation and modes are sophisticated in CFRP-reinforced steel structures. Limited studies have been carried out to investigate the interaction mechanism between nonlinear guided waves and the kissing bond in CFRP-reinforced steel structures, and appropriate nonlinear parameters used for the evaluation of the kissing bond damage should be determined based on the interaction mechanism. Finite element modelling is usually conducive for understanding the interaction between the guided waves and the kissing bond, and the major concern in the modelling is how to simulate the kissing bond properly, so that it can induce nonlinear effects when a guided wave passes through it. Some previous studies have presented various simulation methods for the kissing bond in metal composites. Most of these studies assigned hard contact [25,26] or “seam” [27] to the surfaces of debonding without adhesive properties. To consider changes in the adhesive properties of the bonding layer, which induce nonlinear effects when interacting with guided waves, different methods have been provided. Bu et al. [28] changed the third-order elastic constant of the local or whole adhesive layer to implement the adhesion weakening-induced nonlinear effect. Ginzburg et al. [29] introduced “mechanical diode crack model” to the cohesive FE elements at the interface of the kissing bond. Debonding with a frictionless contact was adopted by both Carrino’s and Brunetti’s research groups, while a bilinear law was set to the contact by Carrino et al. [30], and a cubic contact pressure overclosure relationship was established for the adhesive joint by Brunetti et al. [31].

In our study, the mixed frequency response approach is applied to detect the kissing bond in CFRP-reinforced steel plates based on ultrasonic guided waves. A novel FE simulation method considering adhesive properties with a shear stiffness value close to the actual kissing bond at the contact interface in CFRP-reinforced steel structure is proposed for the investigation of the interaction mechanism between kissing bond and ultrasonic guided waves. And both FE modelling and experiments are carried out to evaluate the effect of the size of the kissing bond on the CAN. Furthermore, in FE modelling and the experiment, excitation wave modes with different wave structures are employed to compare their efficiency for the kissing bond detection in CFRP-reinforced steel structures. This paper is arranged as follows. In Section 2, the principle of mixed frequency response method and the corresponding nonlinear parameter selected for the evaluation of the kissing bond are introduced. Section 3 presents the two groups of excitation wave modes selected based on the dispersion curves and wave structures of a CFRP-reinforced plate. Section 4 describes FE modelling for the kissing bond detection and the results, while Section 5 demonstrates the experiment setup and the analysis of kissing bond detection for specimens with different sizes of damage. Finally, the conclusions of this paper are presented in Section 6.

## 2. Methodology of Mixed Frequency Response Generated by Kissing Bond

The mixed harmonics generated in the composite structure can be explained based on the theoretical analysis for the effects of vibro-acoustic modulation on CAN in composites with delamination [32]. The displacement field u, v and w corresponding to x, y and z direction (as the coordinates in Figure 2) respectively, at the position where CAN occurs can be expressed by

(1) $$\left\{\begin{array}{c} u=\psi_{x}\left(x,y,z\right)p\left(t\right)+\psi_{x0}\left(x,y,z\right)p_{0}\left(t\right)\\ v=\psi_{y}\left(x,y,z\right)p\left(t\right)+\psi_{y0}\left(x,y,z\right)p_{0}\left(t\right)\\ w=\psi_{z}\left(x,y,z\right)p\left(t\right)+\psi_{z0}\left(x,y,z\right)\;p_{0}\left(t\right) \end{array}\right.$$

where $\psi_{i}$ and $\psi_{i0}$(i = x, y, z) are modal functions; p and p_0_ are periodic functions. $\psi_{i}\;\left(x,y,z\right)p\left(t\right)$ stands for the linear displacement and $\psi_{i0}\left(x,y,z\right)p_{0}\left(t\right)$ represents the perturbation.

The existence of a kissing bond in the composite structure introduces CAN when the mixing wave passes through, and at the same time, changes the stiffness of the structure. Considering the excitation force **T** and the nonlinear contact stiffness K_can_, the motion function can be obtained when only the frequency component is focused on

(2) $$M_{1}\ddot{p}\left(t\right)+K_{\alpha }p\left(t\right)=T$$

(3) $$M_{2}{\ddot{p}}_{0}\left(t\right)+K_{\alpha 0}p_{0}\left(t\right)=-K_{\beta }p\left(t\right)$$

In Equation (3), **M_1_** and **M_2_** are two mass submatrices separated from the original mass matrix due to the perturbation; **T** is the excitation vector with two fundamental frequency components ω_1_ and ω_2_. $K_{\alpha }\;$ is related to the transformed stiffness matrix of the composite material and the partial differential modal function vector. $K_{\alpha 0}$ is a constant matrix, which can be obtained using the partial differential perturbation modal function vector and the transformed stiffness matrix of the composites. $K_{\beta }$ can be derived by the partial differential modal function vector and the nonlinear contact stiffness K_can_. From Equation (3), it can be noticed that **p** has the same frequency components as **T**, and the frequency components of $p_{0}$ are equal to those of $-K_{\beta }p\left(t\right)$. If a periodic function $s\left(t\right)$ is set for the kissing bond interface, K_can_ is the relative displacement function of $s\left(t\right)$, and t**_can_** has the same circular frequency as $s\left(t\right)$.

It is assumed that vector **p** contains two sinusoidal signals with circular frequency of ω_1_ and ω_2_ and phases of θ_1_ and θ_2_, respectively, and K_can_ is expressed as follows:

(4) $$K_{can}\left(s\left(t\right)\right)=\frac{k_{\lambda ,0}}{2}+(\sum_{n=1}^{\infty }(k_{\lambda ,n}cos\left(n\omega t\right)+k_{\mu ,n}sin\left(n\omega t\right)))$$

(5) $$k_{\lambda ,n}=\frac{\omega }{\pi }\int_{-\frac{\pi }{\omega_{z}}}^{\frac{\pi }{\omega_{z}}}K_{can}\left(s\left(t\right)\right)cos\left(n\omega t\right)dt,\;\left(n=0,1,2,\ldots \right)$$

(6) $$k_{\mu ,n}=\frac{\omega }{\pi }\int_{-\pi /\omega_{z}}^{\pi /\omega_{z}}K_{can}\left(s\left(t\right)\right)sin\left(n\omega t\right)dt,\;\left(n=1,2,3,...\right)$$

where ω is the greatest common divisor of ω_1_ and ω_2_.

Based on Equations (3) and (4), $-K_{\beta }p\left(t\right)$ can be determined as

(7) $$-K_{\beta }p\left(t\right)=\left[\begin{array}{c} 0\\ 0\\ \begin{array}{c} \frac{\partial^{2}\psi_{z}}{\partial z^{2}}\{\left(\frac{k_{\lambda ,0}}{2}-{\bar{C}}_{33}\right)\left(sin\left(\omega_{1}t+\theta_{1}\right)+\sin\left(\omega_{2}t+\theta_{2}\right)\right)\\ +\frac{1}{2}\sum_{n=1}^{\infty }\sqrt{k_{\lambda ,n}^{2}+k_{\mu ,n}^{2}}(sin\left(\left(\omega_{1}+n\omega \right)t+\theta_{1}-\theta_{n}\right)+sin\left(\left(\omega_{1}-n\omega \right)t+\theta_{1}+\theta_{n}\right)\\ \;\;\;\;+sin(\left(\omega_{2}+n\omega \right)t+\theta_{2}-\theta_{n})+sin\left(\left(\omega_{2}-n\omega \right)t+\theta_{2}+\theta_{n}\right))\} \end{array} \end{array}\right]$$

where $\theta_{n}$ implies the phase induced by CAN, tan$\theta_{n}$ = $k_{\mu ,n}/k_{\lambda ,n}$. From the third element of the matrix in Equation (7), the frequency components of $-K_{\beta }p\left(t\right)$ as well as $p_{0}$ can be figured out. The frequency components can be mainly divided into three groups: (a) the fundamental frequencies ω_1_ and ω_2_; (b) higher harmonic frequencies iω_1_ and iω_2_ (i = 2, 3, 4, …); (c) mixed frequency components ω_2_ ± iω_1_ and ω_1_ ± iω_2_.

It is worth noting that this theory can be used for an arbitrary form of nonlinear contact stiffness K_can_. The frequency components of $p_{0}$ have indicated the nonlinear contact stiffness K_can_ can introduce mixed frequency response and higher harmonics in the composite structures.

To evaluate the severity of kissing bond damage in composite structures, nonlinear parameters are calculated by [33]

(8) $$\beta_{1}=\frac{A_{f_{2}-f_{1}}}{A_{f_{1}}A_{f_{2}}},\;\beta_{2}=\frac{A_{f_{1}+f_{2}}}{A_{f_{1}}A_{f_{2}}},$$

where $f_{1}$ and $f_{2}$ denote two excited fundamental frequency components, $\omega_{1}=2\pi f_{1}$ and $\omega_{2}=2\pi f_{2}$, and $A_{f_{1}}$ and $A_{f_{2}}$ are amplitudes of fundamental frequency components at frequencies of f_1_ and f_2_, respectively. $A_{f_{2}-f_{1}}$ and $A_{f_{1}+f_{2}}$ are amplitudes of mixed frequency components at frequencies of $f_{2}-f_{1}$ and $f_{1}+f_{2}$, respectively.

## 3. Selection of Excitation Wave Mode

**Figure 1:**
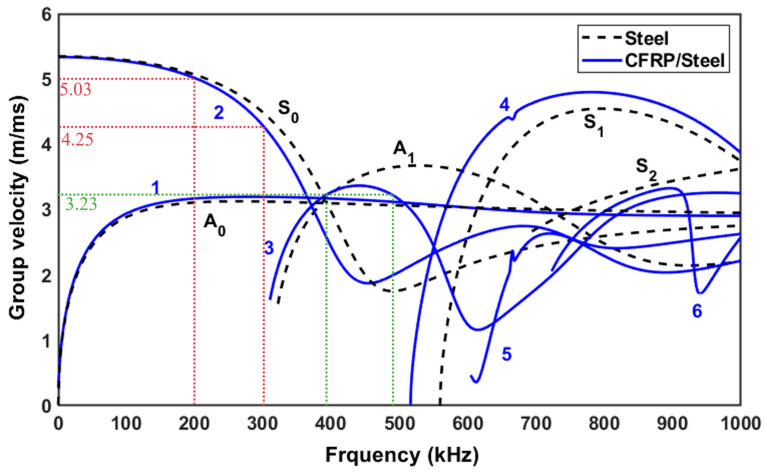
Dispersion curves of CFRP (1 mm)-reinforced steel (5 mm) plate and 5 mm steel plate.

To compare the sensitivity of different wave modes to the kissing bond damage, two groups of excitations were selected based on the dispersion curves of a CFRP-steel plate, in which the CFRP laminate is unidirectional and 1 mm in thickness, while the steel substrate is 5 mm in thickness. The dispersion curves were plotted using software Dispersion Calculator v3.1 [34]. The material properties and the dispersion curve are shown in Table 1 and Figure 1. Dispersion curves of a 5 mm steel plate are also plotted in the figure as a reference. Each group of excitation contains two waves. Wave mode 2 at frequencies of 200 kHz and 300 kHz is selected as Group A excitation, as indicated in Figure 1 as red dashed lines, and Wave mode 3 at frequencies of 390 kHz and 490 kHz is selected as Group B excitation, shown in Figure 1 as green dashed lines. At all these frequencies, mode 2 or mode 3 is the fastest mode, so they can be easily separated from the other modes after propagating a certain distance. It should be noted that in this study only CAN is concerned and to suppress the material nonlinearity of the specimen, the matching of the phase velocity of two waves was not considered during the excitation wave selection [35].

## 4. FE Simulation Analysis

### 4.1. Modelling of Guided Wave-Based Kissing Bond Detection in CFRP-Reinforced Steel Plate

**Figure 2:**
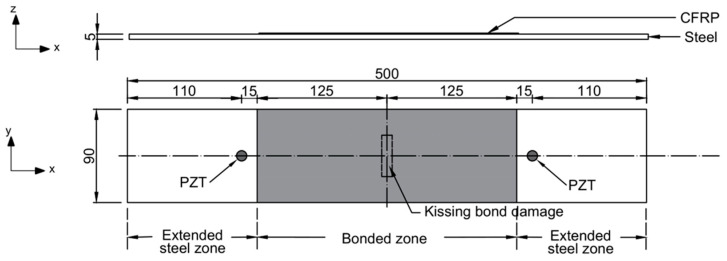
Configuration of the CFRP-reinforced steel plate.

To investigate the interaction between guided waves and the kissing bond in CFRP-reinforced steel plates, the finite element (FE) simulation was carried out. A 3D FE model with element type C3D8R in Abaqus/Explicit was established to simulate the nonlinearity caused by the kissing bond in the CFRP-reinforced steel plate. The dimension of the model, which has the same thicknesses and material properties of CFRP and steel layers as the model for dispersion curve plotting, is shown in Figure 2. Two PZT wafers were placed on the steel plate and were 15 mm away from the CFRP layer. One of them acted as an actuator and another worked as a sensor. To achieve the accuracy of the calculation, the time increment was selected as 4 × 10^−8^ s. For the convergence of the calculation and time saving of computation, the minimum element length was chosen as 0.4 mm from the location of the PZT actuator to the location of PZT receiver, including the CFRP-steel bonded zone and part of the extended steel zone, and for the extended steel zone outside the actuator to sensor monitoring region, the element size in the x direction was changed to 1 mm. The element size of 0.4 mm was determined as less than 1/20 of the wavelength of the received wave mode at the highest frequency of interest (880 kHz) [36], so that the damage-induced nonlinear guided waves could be captured accurately and the multi-scale mesh would not influence the evaluation of the nonlinear effect. The equivalent excitation was simulated as dynamic loads applied in the radial direction at nodes surrounding the circular PZT actuator. Considering the frequency bandwidth of the mixed waves will influence the amplitude of the damage-induced CAN [37], the duration of the excitation signal was thoroughly determined, and finally, 30-cycle Hanning-windowed tone bursts were used for both Group A and Group B excitation. Meanwhile, considering the difference in group velocities of the two waves in each group of excitations, the excitation time of two waves was adjusted to make them overlap when they interact with the kissing bond damage. The time difference of two waves was calculated using the distance from the PZT actuator to the location of the damage divided by their group velocity differences, and these two signals with a time difference were combined and sent to a single PZT actuator. Displacements in the x direction of the model were collected and averaged at the surrounding nodes of the PZT sensor for further signal analysis.

It is worth noting that the bonding condition was simulated as a contact interaction between CFRP and the steel layer instead of introducing a bonding layer in the model, considering the bonding layer is quite thin compared with CFRP and steel layer and it has negligible influence on the propagation of waves. The perfect bonded zone between CFRP and the steel layer was simulated by the “TIE” function in Abaqus. To introduce a kissing bond between the CFRP and steel layers, a surface-to-surface contact interaction was first set at the location of the kissing bond and then a cohesive contact property with appropriate stiffness was assigned to the contact interface. The stiffness was set as 20% of the shear stiffness of the perfectly bonded zone using epoxy (Araldite 420A and 420B, Huntsman, Salt Lake City, UT, USA) and is consistent with the values from the reference [38] for a kissing bond category. To analyze the influence of length of the kissing bond on the mixing waves, five models were established with different kissing bond lengths for both Group A and Group B excitations. The kissing bond damage was located in the middle of the model, as in Figure 2. The length (in the y direction) of it was changed from 0 mm to 40 mm with a 10 mm increment and the width (in x direction) of the damage was kept at 10 mm for all the models.

**Figure 3:**
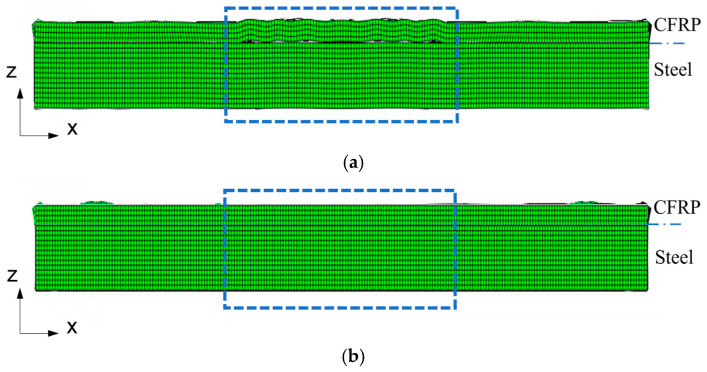
Deformation at the middle cross-section of the simulation model with 30 mm length kissing bond damage when the kissing bond interface between CFRP and steel is (**a**) open at time step of 90 μs and (**b**) closed at time step of 100 μs. The blue rectangle shows the location of the kissing bond at the cross-section. The deformation scale factor is 5 × 10^5^.

Figure 3 shows the deformation of the middle cross-section of the model with a 30 mm length kissing bond damage at two typical time steps. The deformation scale factor is enlarged to show the displacement at the interface more clearly. In these figures, the top layer is CFRP and the bottom is steel. The blue rectangle shows the location of the kissing bond. Figure 3a shows that the interface is open and at this time step the mixing wave would be separated and cannot transmit at the interface, while in Figure 3b, the interface is closed and the mixing wave can pass through the interface. This indicates this model successfully simulated the breathing effect at the interface and as the mixing wave travels through the interface, it would be modulated, causing the change of K_can_ in Equation (4). Since different lengths of kissing bond damage would have different K_can_ values, it would generate different amplitudes of nonlinear response at sum and difference frequencies.

### 4.2. Simulation Results

**Figure 4:**
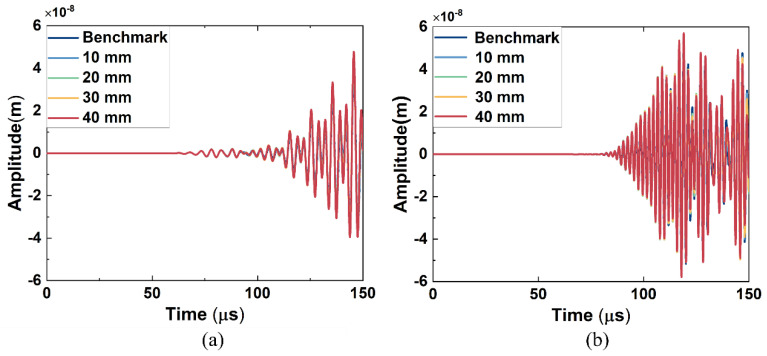
Received time domain signals of FE simulation models with different lengths of kissing bond damage under (**a**) Group A excitation and (**b**) Group B excitation.

**Figure 5:**
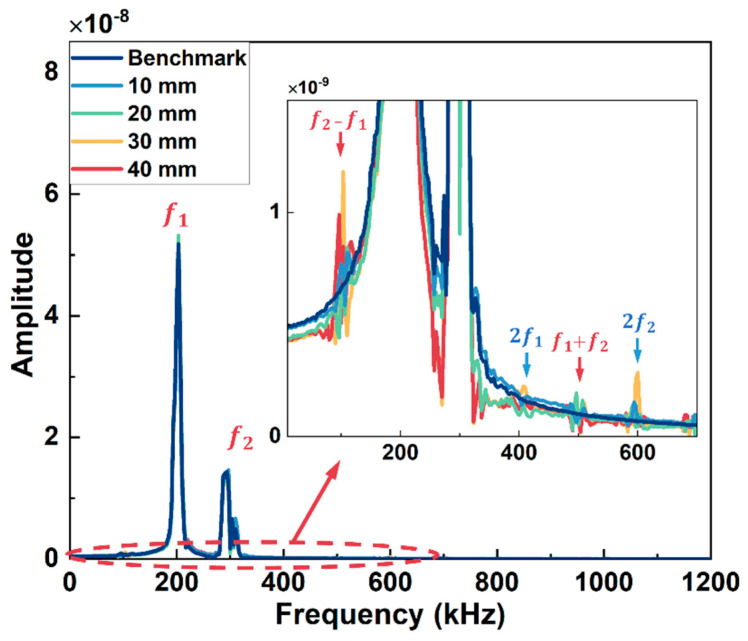
Frequency spectra of simulation models with different lengths of kissing bond damage under Group A excitation. Detailed spectra within the concerned frequency range are zoomed in.

**Figure 6:**
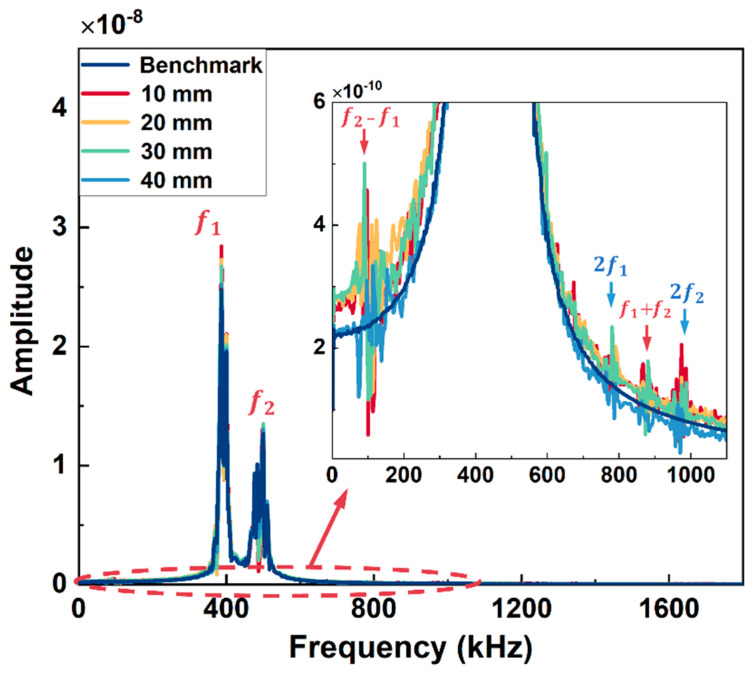
Frequency spectra of simulation models with different lengths of kissing bond damage under Group B excitation. Detailed spectra within the concerned frequency range are zoomed in.

The received signals from the simulation model of Group A and Group B excitation are in Figure 4a and Figure 4b, respectively. In Figure 4a, the arrival time of the first wave packet is around 60 μs, which is close to the theoretical values calculated from the dispersion curve (56 μs for wave mode 2 at 200 kHz), and all the received signals have no significant difference in both amplitude and shape of the wave packet. The arrival time of the first signal is around 82 μs in Figure 4b, which is also consistent with the theoretical values (86 μs for wave mode 3 at 390 kHz), and the signals from models with different lengths of kissing bond damage also have no apparent difference. Thus, the kissing bond damage cannot be identified directly through the time domain signals. Further, the Fast Fourier Transform (FFT) processing method was applied to the received signals and the obtained frequency spectra are shown in Figure 5 and Figure 6. It is worth noting that the received signals before 110 μs for both Group A and Group B excitations were used for the FFT.

In Figure 5, apart from the fundamental frequency components (f_1_ and f_2_) at 200 kHz and 300 kHz, the difference frequency components (f_2_ − f_1_) and sum frequency components (f_1_ + f_2_) appear at 100 kHz and 500 kHz, respectively, for all the models except the model without kissing bond damage. Furthermore, higher-order harmonics (2f_1_ and 2f_2_) are also generated in these models. Similar results are observed in Figure 6 for Group B excitation. The difference frequency components and sum frequency components appear at 100 kHz and 880 kHz, respectively, for all the models with kissing bond damage, along with the higher-order harmonics at 780 kHz and 980 kHz, while these frequency components are absent in the frequency spectrum of the model without damage.

**Figure 7:**
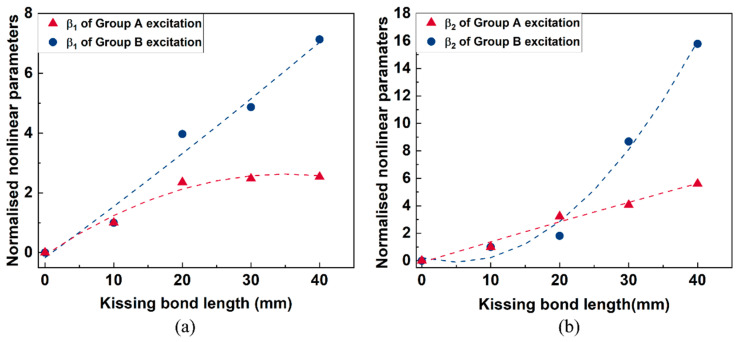
The plot of normalized nonlinear parameters (**a**) β_1_ and (**b**) β_2_ with kissing bond damage length. The dashed lines in these figures are fitting curves.

In the above frequency spectra, the amplitudes of difference and sum frequency components from models with different sizes of kissing bond damage show apparent distinctions. To figure out the capacity to quantify the kissing bond damage length using the mixed frequency response method, amplitudes of difference and sum frequency components were extracted after a series of signal processing on the received signals. First, the Short-Time Fourier transform (STFT) was performed on the received signals. And then the time domain signals at fundamental, difference and sum frequencies were extracted using a band pass filter with a bandwidth of 50 kHz. The amplitudes of first arrived wave packets in these time domain signals were used to calculate the nonlinear parameters based on Equation (8). After calculation, nonlinear parameters for all the models with different lengths of kissing bond damage are plotted in Figure 7. It is observed that for both nonlinear parameters β_1_ and β_2_, as the length of the kissing bond damage changes from 0 to 40 mm, they increase dramatically. In addition, after normalizing the nonlinear parameters for both β_1_ and β_2_, Group B excitation increases faster than Group A excitation, indicating its higher sensitivity to the change of damage length.

**Figure 8:**
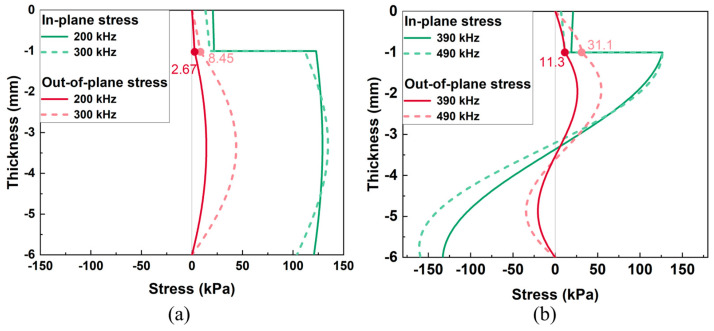
Wave structures of (**a**) Group A excitation and (**b**) Group B excitation. In-plane stress and out-of-plane stress are the stresses in x and z directions, respectively.

To find out the cause of different sensitivity to kissing bond damage, the through thickness wave structures of two groups of excitations were investigated. Figure 8 shows the through thickness stresses of Group A and Group B excitation, respectively. Considering that kissing bond damage is a kind of interfacial damage whose breathing behavior mainly presents in the thickness direction, it would also predominantly modify the passing wave in the thickness direction. Therefore, the wave mode with higher out-of-plane stress would be more sensitive to the damage and thereafter, more energy would be converted to mixed frequency components. As shown in Figure 8, the out-of-plane stresses of Group A excitation are 2.67 kPa and 8.45 kPa at the thickness of 1 mm, where the kissing bond damage is located, while the out-of-plane stresses of Group B excitation are 11.3 kPa and 31.1 kPa, which are much higher than those of Group A excitation. This could be the reason that Group B excitation shows higher sensitivity to the length change of the kissing bond damage.

## 5. Experimental Test

### 5.1. Specimen Preparation and Experiment Setup

**Figure 9:**
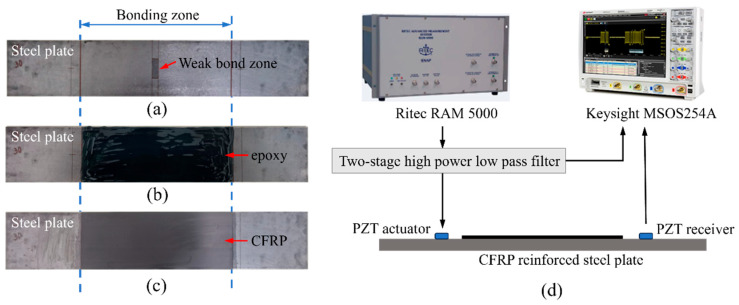
The preparation of the specimen with (**a**) mold release agent brushed at the kissing bond zone, (**b**) epoxy applied for the bonding layer and (**c**) CFRP attached to it. (**d**) The signal generation and acquisition system.

To detect the kissing bond in a CFRP-reinforced steel plate and investigate the effect of damage size on the guided wave nonlinear characteristics, five steel plates reinforced by unidirectional CFRP plates were prepared. One of the steel plates was perfectly bonded with CFRP using epoxy as the benchmark and for the other four specimens, kissing bonds were introduced between the CFRP plates and the steel plates. The artificial kissing bond was induced using a mold release agent, referring to the previous research [39]. After sandblasting the surface of steel plates and cleaning them with acetone, the mold release agent (Loctite 770NC, Henkel, Rocky Hill, CT, USA) was first brushed at the center of the steel plates within the kissing bond regions with different areas, and they were left to stand for a few minutes until the mold release agent dried. Then, epoxy (Araldite 420A and 420B) was applied on the whole bonded zone on the steel plates, followed by the CFRP attached to them. Finally, the specimens were clamped and pressed by heavy objects until the epoxy cured after 48 h. One of the prepared specimens at different stages is shown in Figure 9a–c. The dimensions of all the specimens and damage sizes, as well as the material properties, are the same as the model in the simulation analysis.

The signal generation and acquisition system for the experimental test is shown in Figure 9d. A computer-controlled system ( RAM 5000 SNAP, RITEC, Rochester, NY, USA) designed for the nonlinear ultrasonic technique was used to generate signals, which were two 30-cycle Hanning-window modulated sinusoidal mixing tone bursts, and were sent to a PZT actuator (diameter 10 mm), exciting guided waves in the specimen. A specific time difference between two mixing tone bursts was set so that they can synchronously interact with the kissing bond damage. Both Group A and Group B excitations were adopted in the experimental test. The PZT actuator was glued on the surface of the steel plate at one end with a 15 mm distance to the CFRP and another PZT wafer (diameter 10 mm) as a receiver was glued at the other end of the steel plate, as shown in Figure 9d. Wave signals acquired by the PZT receiver were averaged 1024 times to minimize the environmental noise and were then recorded by the oscilloscope (MSOS254A, Keysight, Santa Rosa, CA, USA) at a sampling frequency of 20 MHz. All the received signals were further denoised using a high-pass filter in MATLAB 2021 to remove the low-frequency environmental noises and were processed using the FFT to obtain the frequency spectrum.

### 5.2. Experiment Results

**Figure 10:**
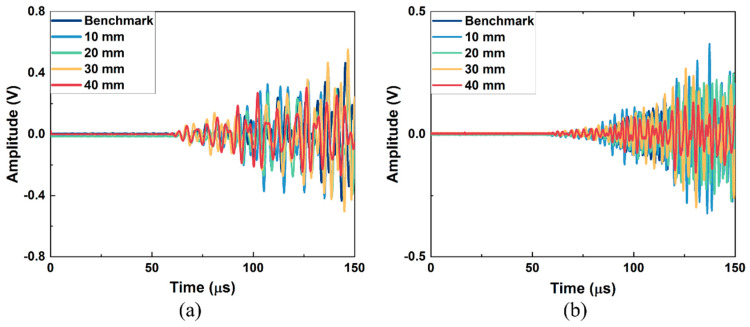
The experimental received signals from specimens with different lengths of kissing bond damage under (**a**) Group A excitation and (**b**) Group B excitation.

The received signals after denoising in the time domain are shown in Figure 10. For both Group A and Group B excitation, the received signals were complex and the damage information was difficult to extract from the time domain signal directly. Although the shapes of the waveform are different from those in the simulation results due to the influence of the epoxy layer on the wave propagating in the experiment, the arrival times of the first waves are at 60 μs for Group A excitation and at around 80 μs for Group B excitation, which is close to the simulation results. For both groups of excitations, the signal shows obvious differences in the waveforms and amplitudes among specimens with different damage lengths. However, considering five specimens were used for different kissing bond damages and the benchmark, and considering that our excitation involved mixing waves with long duration, part of the received signal differences could be caused by the deviation of the specimen manufacturing and the attaching of PZTs. Thus, it cannot be confidently concluded that the differences in the linear features of the signals are completely caused by the damage. Further processing methods were required to extract the damage information from the received signals and to realize the quantitative damage evaluation.

**Figure 11:**
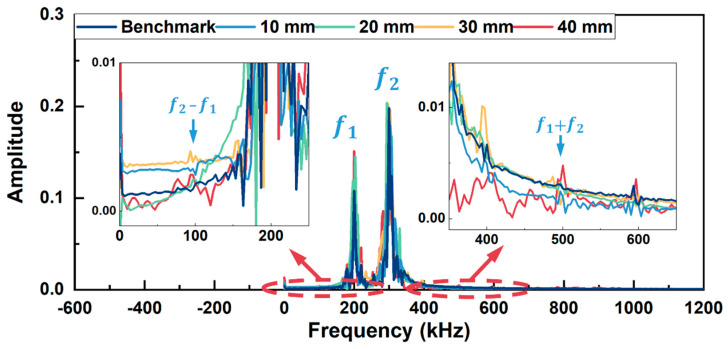
Frequency spectra of the received signals from specimens with different lengths of kissing bond damage under Group A excitation.

**Figure 12:**
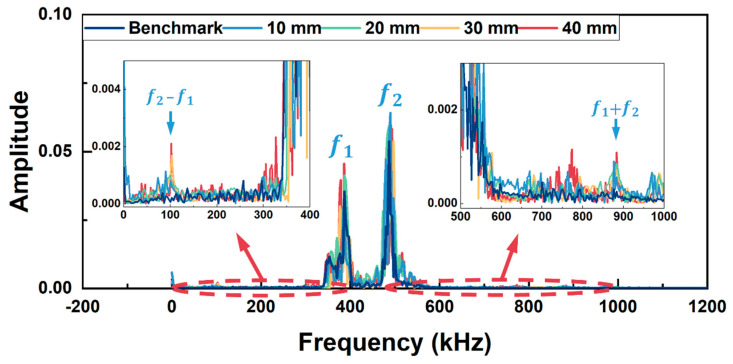
Frequency spectra of the received signals from specimens with different lengths of kissing bond damage under Group B excitation.

FFT spectra in Figure 11 and Figure 12 were obtained for these received signals. In the frequency spectrum for Group A excitation (in Figure 11), sidebands at sum frequency (500 kHz) and difference frequency (100 kHz) appear beside two fundamental frequencies (200 kHz and 300 kHz) for the specimens with kissing bond damages. Meanwhile, higher-order harmonics can also be observed in the frequency spectrum, including second harmonics at 400 kHz and 600 kHz. As a comparison, the signal from the specimen without kissing bond damage shows no apparent sum or difference frequency components, while the second harmonics at 400 kHz and 600 kHz are obvious. These second harmonics are probably due to the nonlinearity from the measurement system. For the Group B excitation case (in Figure 12), similar results can be observed. For the specimens with kissing bond damages, sum and difference frequency components appear at 100 kHz and 880 kHz. Second harmonics at 780 kHz and 980 kHz can also be seen in the spectra. However, in the intact specimen, sum or difference frequency components are hardly noticeable. These results reveal that using the mixed frequency response method can detect the kissing bond damage in composite structures, and this method is a more preferred option than the higher harmonic generation method when the detection system is interfered with by the system nonlinearity, which conceals the damage-induced CAN.

**Figure 13:**
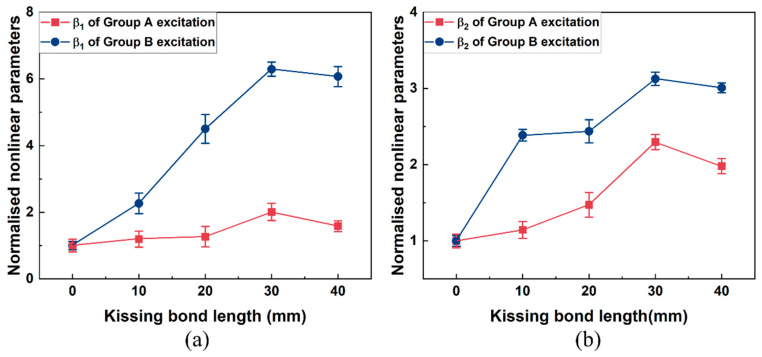
Nonlinear parameters (**a**) β_1_ and (**b**) β_2_ with kissing bond lengths.

It is also found out that the amplitudes of sum or difference frequency components generated by various sizes of kissing bond damage are different. With further STFT processing on the received signals, time domain signals at fundamental and sum and difference frequencies were extracted and the nonlinear parameters were calculated using Equation (5) based on the amplitudes of the first arriving wave packets in these signals. To compare the sensitivity of different excitation wave modes on the kissing bond damage detection, the nonlinear parameters from Group A and Group B excitation were normalized and plotted with the length of the kissing bond damage in Figure 13. For each kissing bond length, three specimens were made and the same experimental test and signal processing method were applied on them to verify the repeatability of the experiment results. The variations of these results are shown as error bars in Figure 13.

In Figure 13, nonlinear parameters β_1_ and β_2_ change positively with the kissing bond length from 0 mm to 30 mm for both Group A and Group B excitation cases. The nonlinear parameters reflect the extent of nonlinearity caused by the breathing effect of the interface between the CFRP and the steel plate. As the length of the kissing bond increases, the area of the breathing interface is enlarged and the forces of the open and close action at the interface become more significant, which in turn makes the nonlinear response stronger and the nonlinear parameter higher. Nevertheless, the increasing trend changes at 30 mm damage length, demonstrating a decrease in the nonlinearity, which is different from the simulation result. This phenomenon could be caused by the decline in the breathing effect of the interface in the experiment specimen due to the overlong size of the damage. In the simulation model, the breathing effect always exists due to the fixed interface properties, while in the experiment, when the damage in the specimen is too long, the whole interface is difficult to contact intimately after introducing the kissing bond damage and therefore, the breathing effect would be weakened under the same excitation voltage level. The decrease in nonlinearity can also be proved by the change in the linear characteristics of the guided waves. For example, in Figure 13, an obvious drop in the amplitude of the signals from 40 mm damage can be observed for both Group A and Group B excitations, compared with the signals with other sizes of damage. The inconsistent results indicate that the simulation of the interface still has differences from the real condition. Further analysis involving the interface behavior change with different damage length and the impact of interface properties like friction on the breathing behavior should be considered in the kissing bond damage simulation. In addition, the results in Figure 13 show that the variation of nonlinear parameters from Group B excitation is more significant than that from Group A, which is similar to the simulation results. The results again confirm that the excitation wave mode with higher stress in the thickness direction is more sensitive to the kissing bond damage between the CFRP and steel plates. When the wave travels through the interface, the breathing effect caused by the damage is dominantly out of plane. Therefore, the wave mode with higher out-of-plane stress is more sensitive to the nonlinearity caused by the kissing bond damage.

## 6. Conclusions

The mixed frequency response method was successfully used to detect kissing bond damage in CFRP-reinforced steel plates through both FE simulation and experiment. Nonlinear parameters were adopted to quantitatively evaluate the damage length up to 40 mm. In simulation, a cohesive interface was considered creatively to analyze the interaction between the interface of the kissing bond and the guided waves. The breathing effect was efficiently simulated at the interface using the proposed method. The simulation results show that the nonlinear parameter increases dramatically with the length of the damage. The experiment results were similar to those of simulation results, but when the damage length reached 30 mm, the nonlinearity caused by kissing bond damage gradually decreased and this damage can be directly detected by the linear characteristic of guided waves. Furthermore, in this study excitation wave modes with different out-of-plane stress levels were innovatively compared for their sensitivity to the kissing bond damage. The results showed that wave modes with higher out-of-plane stress were more sensitive to the damage, revealing the importance of excitation wave mode selection for kissing bond damage detection.

It should be noted that this study still possesses some limitations. During frequency domain analysis, the selection of the time window length presents a certain degree of influence on the values of nonlinear parameters. When employing different designs of test specimens and different excitation frequencies, the length of the time window must be carefully determined to ensure the stability and reliability of results. Meanwhile, due to the high sensitivity and subtlety of nonlinearity, multiple measurements in experimental tests are necessary to guarantee the repeatability of the results. In addition, this study did not consider the influence of nonlinear guided wave propagation distance and damage locations on the quantitative evaluation of kissing bond damage. To minimize the impact of these parameters, a relatively short detection distance for the nonlinear waves and a consistent damage location were adopted for all specimens with different damage lengths. Regarding the shear stiffness for the contact interface in the simulation, only empirical values from previous studies were adopted, which may deviate from the actual condition of experimental specimens. However, since this study mainly focused on verifying whether the newly proposed contact interface simulation method can successfully simulate the breathing behavior, which could induce nonlinear effects, and the simulated interface indeed demonstrated the breathing behavior, the preliminary shear stiffness values do not influence the conclusions of this study. Further studies will be carried out for the impact of various parameters, such as propagation distance, damage location and shear stiffness at the simulated interface, on the quantitative assessment of kissing bond damage. To better align the simulation model with real-world conditions, a more comprehensive modelling of the contact interface is required. Factors like friction at the interface should be considered. The detection of guided waves using combined linear and nonlinear methods of guided waves will also be investigated to accomplish the detection of different stages of the debonding in CFRP-reinforced steel structures.

## Figures and Tables

**Table 1:** Material properties of CFRP and steel.

**Steel**	**Density (kg/m^3^)**			**Young’s modulus (GPa)**				**Poison’s ratio**		
	7850			200				0.33		
**CFRP**	Density (kg/m^3^)	E_1_ (GPa)	E_2_ (GPa)	E_3_ (GPa)	Nu_12_	Nu_13_	Nu_23_	G_12_ (GPa)	G_13_ (GPa)	G_23_ (GPa)
	1600	41	6.6	6.6	0.33	0.33	0.5	7.3	7.3	2.2

## Data Availability

The original contributions presented in this study are included in the article. Further inquiries can be directed to the corresponding author.
